# Population-based incidence of Type 2 diabetes and its associated risk factors: results from a six-year cohort study in Iran

**DOI:** 10.1186/1471-2458-9-186

**Published:** 2009-06-16

**Authors:** Hadi Harati, Farzad Hadaegh, Navid Saadat, Fereidoun Azizi

**Affiliations:** 1 Prevention of Metabolic Disorders Research Center, Research Institute for Endocrine Sciences, ShahId Beheshti University(M.C), Tehran, Iran

## Abstract

**Background:**

The Middle East is estimated to have the largest increase in prevalence of diabetes by 2030; yet there is lack of published data on the incidence of Type 2 diabetes in this region. This study aimed to estimate Type 2 diabetes incidence and its associated risk factors in an Iranian urban population.

**Methods:**

Among 3307 non-diabetics ≥ 20 years (mean age 42 ± 13 years, 42% males), glucose tolerance test was performed at baseline in 1999–2001 and at two consecutive phases in 2001–2005 and 2005–2008. Diabetes and glucose tolerance status were defined according to the ADA 1997 criteria. Logistic regression was used to determine the independent variables associated with incident diabetes and their odds ratios (OR).

**Results:**

After median follow-up of 6 years, 237 new cases of diabetes were ascertained corresponding to an age and sex standardized cumulative incidence of 6.4% (95%CI: 5.6–7.2) and incidence rate of 10.6 (9.2–12.1) per 1000 person years. Besides classical diabetes risk factors, female sex and low education level significantly increased risk of diabetes in age adjusted models. In full model, the independent predictors were age [OR, 95%CI: 1.2 (1.1–1.3)], family history of diabetes [1.8 (1.3–2.5)], body mass index ≥ 30 kg/m^2 ^[2.3 (1.5–3.6)], abdominal obesity [1.9 (1.4–2.6)], high triglyceride [1.4 (1.1–1.9)], Isolated impaired fasting glucose (IFG) [7.4 (3.6–15.0)], Isolated impaired glucose tolerance (IGT) [5.9 (4.2–8.4)] and combined IFG and IGT [42.2 (23.8–74.9)].

**Conclusion:**

More than 1% of the Iranian urban population older than 20 years develops Type 2 diabetes each year. Combination of IFG and IGT was the strongest predictor of incident diabetes among the modifiable risk factors.

## Background

Type 2 diabetes has reached epidemic levels in most populations and epidemiological evidence suggests that unless effective preventive measures are implemented, the prevalence continue to raise globally [1] It is estimated that by the 2030 the number of people with diabetes will increase to more than 366 million, more than twice the number in 2000 [2]. Most of these new cases are from developing countries and it seems that the Middle East is among the regions that will have the largest increase in prevalence of diabetes by 2030 [3]. The occurrence of rapid and major lifestyle changes in these countries has increased the prevalence of obesity and other non-communicable disease risk factors (like hypertension and dyslipidemia) which have been reported to be the major etiologic factors behind the rising incidence of type 2 diabetes around the globe [4]. Identification of these risk factors is the most important step in development of preventive strategies in any population because it has been shown that reduction of such risk factors may reduce the incidence of Type 2 diabetes [5].

The prevalence of diabetes in the Middle Eastern countries is known to be high. It is reported to be 29% in United Arab Emirate [6], and 16.1% in Oman [7]. Recent national survey in Iran showed the prevalence to be 7.7% in subjects 25–65 years [8] and it has been reported to be even higher in the capital city, Tehran (14.0%) [9]. However no report about the incidence of Type 2 diabetes has yet been published in these populations. We aimed to determine the incidence of type 2 diabetes and its associated risk factors by using standardized oral glucose tolerance test (OGTT) in a large population based study in a representative sample of the population of Tehran called the Tehran Lipid and Glucose Study (TLGS).

## Methods

### Study population

**Figure 1:**
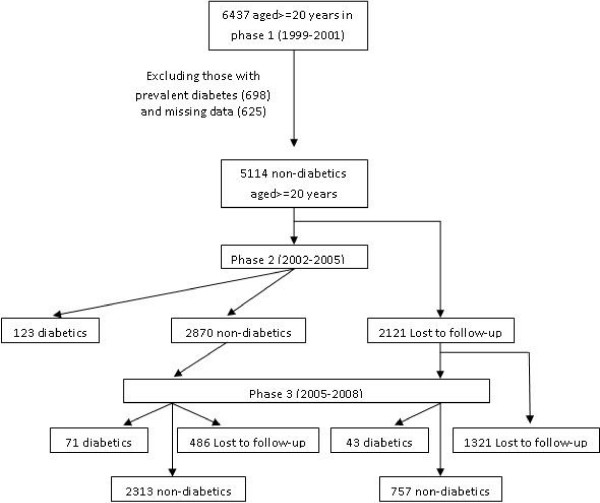
**Follow-up status of the TLGS participants after the baseline examination**.

The TLGS is a prospective population based study performed on a representative sample of the Tehran population, with the aim of determining the prevalence of non-communicable disease (NCD) risk factors and developing a healthy lifestyle to improve them [10]. The baseline survey was performed from 1999 to 2001 and 4751 families which included more than 15000 residents of district 13 of Tehran aged over 3 years were selected by cluster random-sampling method. After this cross-sectional prevalence study of NCD risk factors, subjects entered into a cohort and a prospective interventional study. The cohort group constituted of 6437 subjects over 20 years. After exclusion of subjects with prevalent diabetes at baseline (n = 698) and those with missing data regarding fasting and 2-hours glucose (n = 625), there were 5114 non-diabetic subjects in the cohort group which were reexamined in 2 consecutive phases, one from 2002 to 2005 (phase 2) and another from 2005 to 2008 (phase 3) (Figure 1). Those who developed diabetes in the follow-up examinations (phase 2 or 3) and those who completed the phase 3 examination were included in the current study. The main reasons for lack of attendance at follow-up examinations despite repeated calls were either migration or other personal reasons. The proposal of this study was approved by the research council of The Research Institute for Endocrine Sciences of Shahid Beheshti University (M.C) and informed written consent was obtained from each subject.

### Clinical, anthropometric, and laboratory measurements

Subjects were interviewed privately, face-to-face by trained interviewers using pretested questionnaires. Initially, information on demographics, education, smoking status, and medical and drug history was collected. Anthropometric measures including weight, height, waist circumference (WC) was measured according to a standard protocol [10]. Body mass index (BMI) was calculated as weight in kg divided by height in m^2^. Systolic and diastolic blood pressures were measured twice in a seated position in the right arm and the mean value was considered as the subject's blood pressure. A blood sample was taken after 12–14 h overnight fasting and was centrifuged within 30–45 min of collection. All blood analyses were performed at the TLGS research laboratory on the day of blood collection. For OGTT, 82.5 g glucose monohydrate solution (equivalent to 75 g anhydrous glucose) was administered orally to subjects and a blood sample was taken 2 hours later. The analysis of samples was performed using a Selectra 2 auto-analyzer (Vital Scientific, Spankeren, Netherlands). Fasting and 2-hours plasma glucose (FPG and 2 hPG respectively) were measured by enzymatic colorimetric method using glucose oxidase kit (Pars Azmoon Inc., Tehran, Iran); inter-and intra-assay coefficient of variations (CV) were less than 2.2%. For lipid measurements, total cholesterol and triglyceride kits (Pars Azmoon Inc., Tehran, Iran) were used. Triglycerides (TGs) were assayed using enzymatic colorimetric assay with glycerol phosphate oxidase. HDL-cholesterol (HDL-C) was measured after precipitation of the apolipoprotein B containing lipoproteins with phosphotungistic acid. All samples were analyzed when internal quality control met the acceptable criteria. Inter- and intra-assay coefficients of variation were 2 and 0.5% for HDL-C and 1.6 and 0.6% for triglyceride respectively. The glucose measurements were conducted by the same method at the baseline and follow-up examinations and inter-and intra-assay CVs for the follow-up examinations were less than 3.3%.

### Definition of variables and outcomes

Education was categorized into 3 groups: illiterate/primary school, high school and diploma and higher. Positive family history of diabetes was defined as having at least one parent or sibling with diabetes. History of cardiovascular disease (CVD) was defined as previous ischemic heart disease and/or cerebrovascular accidents. We used 1997 American Diabetes Association (ADA) definitions of diabetes (FPG ≥ 7.0 mmol/l, 2 hPG ≥ 11.1 mmol/l or taking of anti-diabetic medication), isolated impaired glucose tolerance (IGT, 2 hPG 7.8–11 mmol/l and FPG<6.1 mmol/l), isolated impaired fasting glucose (IFG, FPG 6.1–6.9 and 2 hPG<7.8 mmol/l) and combined IFG and IGT (IFG/IGT, FPG 6.1–6.9 and 2 hPG 7.8–11 mmol/l)[11]. Type 2 diabetes risk factors were defined as follows: obesity: BMI<25 (normal weight), 25–29.9 (overweight) and ≥ 30 kg/m^2 ^(obese); abdominal obesity: WC>88 or >102 cm in females and males respectively; hypertension: blood pressure ≥ 140/90 and/or taking of antihypertensive medication; high TG: Triglyderide>2.2 mmol/l; Low HDL: HDL-C<1.0 or <1.3 mmol/l in males and females respectively [12].

### Statistical Analyses

Baseline characteristics of the participants and non-participants in the follow-up examination were compared by Chi-square, student t and Mann-Whitney test as appropriate. Sampling weights, which accounted for the unequal probabilities of selection resulting from the complex design and non-response adjustment factors based on Iranian census bureau data (2006) on age and gender, were incorporated to all of the estimation processes. Cumulative incidence of diabetes with 95% (CI) was calculated for each risk factor category by dividing the number of new cases of Type 2 diabetes (drug treated and newly diagnosed) to the total number of subjects in that group. Incidence rate of diabetes was also calculated for the entire cohort by dividing the total number of incident cases to the total person-years (PY) of follow-up. Age and sex standardized incidence was estimated by direct method using the 2006 population data from the national census bureau. Cumulative incidences were compared by Chi-square test. The association of different categorical risk factors (except age) with incident diabetes was first assessed by calculating age adjusted odds ratios (ORs) with 95% CI using logistic regression analysis. The potential risk factors were age, sex, current or past smoking, education level, family history of diabetes, history of CVD, hypertension, BMI 25–29.9 and ≥ 30 kg/m^2^, abdominal obesity, IFG, IGT, IFG/IGT and dyslipidemia (high TG and Low HDL-C). For risk factors with more than 2 categories the first category was considered as the reference group and the p-value was calculated for linear trend in the risk. Those risk factors with a P value less than 0.2 in bivariate analysis were selected to enter into the multivariate model and the final model was constructed by backward stepwise method with P values of 0.1 set as the significance level for removal from the model. Statistical analysis was performed by using STATA version 9.0. Two sided P values less than 0.05 was considered as statistically significant.

## Results

The baseline characteristics of the study population are presented in Table 1. The mean age and BMI of study population were 42 years and 26.7 kg/m2 respectively. Overall, 3307 individuals from the 5114 non-diabetics at baseline either developed diabetes or completed the phase 3 examination (Figure 1). The median follow-up duration was six years. Comparison of responders and non-responders showed that the formers had higher baseline family history of diabetes, diastolic blood pressure, BMI, WC and TG. No significant difference was found between the two groups in baseline age, systolic blood pressure, FPG, 2 hPG and HDL-C (Table 1).

**Table 1:** Comparison of baseline characteristics between respondents and non-respondents in the TLGS cohort*.

	Respondent (N = 3307)	Non-respondent (N = 1807)	P
Age (years)	42 (13)	41 (15)	0.8
Sex (% males)	42.0	43	0.2
Family history of diabetes (%)	27	24	**0.006**
Systolic blood pressure(mm/Hg)	118 (18)	118 (18)	0.9
Diastolic blood pressure(mm/Hg)	78 (10)	77 (11)	**0.02**
Waist circumference (cm)	88 (12)	87 (12)	**0.01**
Body mass index (kg/m^2^)	26.7 (4.7)	26.3 (5.2)	**0.004**
Fasting plasma glucose (mmol/l)	5.0 (0.5)	4.9 (0.5)	0.3
2-hours plasma glucose (mmol/l)	5.9 (1.6)	5.9 (1.6)	0.8
Triglyceride (mmol/l)	1.6 (1.2)	1.5 (1.1)	**0.01**
HDL-cholesterol (mmol/l)	1.0 (0.2)	1.0 (0.2)	0.5

*Respondents were those who developed diabetes during the follow-up or completed the phase 3 examination. Non-respondents did not participate in phase 3 examination.

Data are mean (SD) for continuous (median with inter-quartile range for Triglyceride) and % for categorical variables.

Overall, 237 new cases of Type 2 diabetes were identified after a median follow-up of six years which resulted in a crude cumulative incidence 7.2 (95%CI: 6.3–8.1). The age and sex standardized cumulative incidence was 6.4 (95% CI: 5.6–7.2). The crud and adjusted incidence rate were 11.9 (10.5–13.6) and 10.6 (9.2–12.1) per 1000 PY respectively. Table 2 reports the cumulative incidence and ORs of type 2 diabetes stratified by different risk factors. Risk of diabetes was 30% higher in females after age adjustment. The incidence increased as the subjects got older and reached a plateau in 65 years, after which it did not increase further (cumulative incidence of 12.1 vs. 12.6% in 50–64 and ≥ 65 years age group respectively, P = 0.7). The highest increase in incidence was detected in the 35–49 years group in which it increased nearly 2.5 times compared to the younger group (20–34 years). Most of the 237 incident cases belonged to the 35–49 years group (37.1%) followed by 50–64 (36.3%), 20–34 (15.6%) and ≥ 65 (11.0%) years. The mean age at diagnosis of diabetes was 48(12) years. Incidence of diabetes was higher in those with past history of CVD; however this association was lost after age adjustment. Current or past smoking did not increase the incidence of diabetes. Higher education significantly decreased incidence of diabetes and those with diploma or higher degrees had 50% lower risk of developing diabetes than those who were illiterate or had primary school certificate. As expected, positive family history of diabetes, BMI 25–29.9 and ≥ 30 kg/m2, abdominal obesity, hypertension, high TG, low HDL-C, IFG, IGT and IFG/IGT were all significant predictors of incident diabetes in the age adjusted model with ORs ranging from 1.4 to 42.2. In age adjusted models, the rate of conversion to Type 2 diabetes in individuals with IFG, IGT and IFG/IGT was 8.3, 7.1 and 42.2 times higher than that of subjects with normal FPG and 2 hPG respectively.

**Table 2:** Incidence rate and risk of Type 2 diabetes stratified by different risk factors.

Risk factors	Number	Incident diabetes	Cumulative incidence (%, 95% CI)	Odds ratio (95% CI) *	P
Sex					0.04
Males	1386	91	6.6 (5.3–8.0)	1	
Females	1921	146	7.6 (6.4–8.8)	1.3 (1.0–1.7)	
All	3307	237	7.2 (6.3–8.1)		
					
Age (years)					<0.001
20–34	1108	37	3.1 (2.4–4.6)	1	
35–49	1210	88	7.3‡ (5.9–8.9)	2.4 (1.6–3.6)	
50–64	711	86	12.1†(9.8–14.7)	4.3 (2.9–6.3)	
≥ 65	206	26	12.6 (8.4–17.9)	4.5 (2.6–7.5)	
					
Family history of diabetes					<0.001
No	2335	129	5.6 (4.6–6.5)	1	
Yes	882	99	11.2‡ (9.2–13.5)	2.4 (1.8–3.2)	
					
History of CVD					0.3
No	3119	215	6.9 (6.0–7.8)	1	
Yes	113	16	14.2† (8.3–22.0)	1.3 (0.7–2.3)	
					
Smoking					0.7
Never	2650	189	7.1 (6.2–8.23)	1	
Current	361	30	7.2 (5.7–11.7)	1.0 (0.7–1.6)	
Past	193	18	7.3 (5.6–14.3)	0.7 (0.4–1.2)	
					
Education					0.001
Illiterate/primary school	972	119	12.2 (10.2–14.5)	1	
Secondary school	593	41	6.9‡ (5.0–9.3)	0.8 (0.5–1.2)	
Diploma and higher	1740	77	4.4† (3.5–5.5)	0.5 (0.4–0.8)	
					
Hypertension					<0.001
No	2657	147	5.5 (4.7–6.5)	1	
Yes	631	88	14.0‡ (11.3–16.9)	1.9 (1.4–2.6)	
					
Obesity					<0.001
Normal weight	1190	41	3.5 (2.5–4.6)	1	
Overweight	1346	86	6.4‡ (5.1–7.8)	1.7 (1.1–2.5)	
Obese	771	110	14.3‡ (11.9–16.9)	4.0 (2.7–5.8)	
Abdominal obesity					0.001
No	2225	98	4.4 (3.6–5.3)	1	
Yes	1038	135	13.0‡ (11.0–15.2)	1.4 (1.3–1.5)	
High Triglyceride					<0.001
No	2449	136	5.6 (4.7–6.5)	1	
Yes	858	101	11.8‡ (9.7–14.1)	2.0 (1.5–2.6)	
Low HDL-cholesterol					0.04
No	1001	61	6.1 (4.7–7.8)	1	
Yes	2306	176	7.6‡ (6.6–8.8)	1.4 (1.0–1.9)	
Glucose tolerance category					
Normal	3216	94	2.9 (2.4–3.6)	1	<0.001
Isolated IFG	60	12	20.0 (10.8–32.3) ‡	8.3 (4.2–16.5)	
Isolated IGT	442	85	19.2 (15.7–23.2)	7.1 (5.1–9.8)	
IFG/IGT	77	46	59.7 (47.9–70.8) ‡	42.2 (25.8–75.7)	

*Cumulative incidences were compared by Chi-squared test. Odds ratios were calculated by logistic regression analysis after controlling for age. For categorical variables with more than 2 values, the first category was considered as the reference and the p-value is for linear trend in risk.

Obesity: BMI<25 (normal), 25–29.9 (overweight) and ≥ 30 kg/m^2 ^(obese), Abdominal obesity: waist circumference>88 or >102 cm in females and males respectively, Hypertension: Blood pressure ≥ 140/90 and/or taking of antihypertensive medication, High TG:Triglyderide>2.2 mmol/l, Low HDL: HDL-cholesterol <1.0 or <1.3 mmol/l in males and females respectively, Isolated IFG: FPG 6.1–6.9 and 2 hPG<7.8 mmol/l, Isolated IGT:FPG<6.1 and 2-hours plasma glucose 7.8–11 mmol/l, IFG/IGT: FPG 6.1–6.9 and 2 hPG 7.8–11 mmol/l.

† and ‡ represent P < 0.05 and P < 0.001 in comparison to the preceding category.

All the variables with p value of less than 0.2 in the age adjusted model, namely age, sex, family history of diabetes, education level, BMI 25–29.9 and ≥ 30 kg/m2, abdominal obesity, hypertension, high TG, low HDL-C, IFG, IGT and IFG/IGT were selected to enter into the final model. There was no interaction between sex and any other selected variables; hence no separate analysis was performed for men and women. Categories of BMI and abdominal obesity were fitted separately into the model to prevent co-linearity. However, since the calculated ORs and P values for all the other variables in the final model were quite identical whether BMI or abdominal obesity was in the model and because abdominal obesity fitted models had higher overall fitness in Hosmer-Lemeshow test (Chi-square value of 4.1 vs. 9.6), only calculated ORs of the model that included abdominal obesity were presented for those variables. For age, OR was calculated per 10 years increase in its value. Table 3 demonstrates the result of the final logistic regression model with backward stepwise approach. The highest OR among independent variables was with IFG/IGT (42.2), followed by IFG (7.4), IGT (5.9), BMI ≥ 30 kg/m^2 ^(2.3), abdominal obesity (1.9), family history of diabetes (1.8), hypertriglyceridemia (1.4), and age (1.2).

**Table 3:** Independent variables associated with incident Type 2 diabetes and their corresponding odds ratios in the TLGS cohort population after median follow-up time of 6 years.

Variables	Odds ratio (95%CI)	P
Age (per 10 years)	1.2 (1.1–1.3)	0.008
Family history of diabetes	1.8 (1.3–2.5)	<0.00
BMI ≥ 30 kg/m^2^	2.3 (1.5–3.6)	<0.001
Abdominal obesity	1.9 (1.4–2.6)	0.001
High triglyceride	1.4 (1.1–1.9)	0.04
Glucose tolerance category		
Normal	1	-
Isolated IFG	7.4 (3.6–15.0)	<0.001
Isolated IGT	5.9 (4.2–8.4)	<0.001
IFG/IGT	42.2 (23.8–74.9)	<0.001

Odds ratios were obtained by multivariate logistic regression analysis with backward selection. The variables that entered into the model at first step were age, sex, education level (Illiterate/primary, secondary, diploma/higher), family history of diabetes, hypertension (blood pressure ≥ 140/90 mm/Hg and/or taking of antihypertensive medication), obesity (BMI<25, 25–29.9 and ≥ 30 kg/m^2 ^with <25 as reference group), Abdominal obesity (waist circumference>88 or >102 cm in females and males respectively), glucose tolerance categories, high triglyceride (>2.2 mmol/l) and low HDL cholesterol (<1.0 or <1.3 mmol/l in males and females respectively). BMI categories and abdominal obesity were fitted separately into the models.

## Discussion

In this first report of the population based incidence of diabetes in the Eastern Mediterranean region, which used FPG and 2 hPG to ascertain glucose tolerance status both at baseline and at follow-up, we estimated the standardized incidence rate of Type 2 diabetes in a representative sample of Tehranian adults over 20 years to be 10.6/1000 PY which corresponded to an annual incidence rate of more than one percent. We also showed that among modifiable risk factors namely obesity, hypertriglyceridemia and abnormal glucose tolerance; IFG/IGT was the most prominent determinant of progression to incident diabetes in the Iranian population.

The incidence rate of Type 2 diabetes in most of the European studies that used similar criteria for classification of glucose tolerance ranges from 7.6 to 10.8/1000 PY [13-15]. In particular, in the Ely study in UK the crude incidence rate was 7.5 [14], similar to a recent report in the Australia (7.0/1000 PY) [16] which are lower than our results. All of these European populations were older than ours and none had lower mean BMI. High incidence rate of diabetes has been reported in Netherland in an old population with mean age of 60 years [17]. The reported incidence in the current study is lower than similar population based studies in the US with incidence rate of 11.4–12.8/1000 PY [18-20]. One possible explanation for this observation is that our population is relatively young (mean age of 42 years) compared to these studies. The total and urban population of IRAN in 2006 was more than 70 and 48.2 million respectively, 64% of which were over 20 years old. If we extrapolate the standardized annual rate of the incident diabetes in the present study to the current urban population of IRAN older than 20 years, it is estimated that each year more than 310 thousands individuals develop Type 2 diabetes. This alarming rate confirms previous estimates that the Middle East region will have the highest increase in number of diabetes cases in the following 20 years [21]. This could be due to changes in life style including dietary habits and physical activity pattern that has occurred quite rapidly in recent years due to rapid urbanization and technological transition and has led to a rapid rise in risk factors of chronic disease [22]. High incidence rate of type 2 diabetes in our relatively young population might also be due to ethnicity which is a known risk factor for diabetes [4].

Identification of those who may be at higher risk of developing diabetes is usually the first step for the prevention of Type 2 diabetes. In the present study, age was a significant predictor of the incidence in multivariate analysis. We also found that the highest proportion (37.1%) of incident cases belonged to the 35–49 years age group. This is in accordance with a recent consensus statement from the International Diabetes Federation which states that the age of onset of diabetes has moved down into younger adults [1]. Similar to the results of a recent Australian study [16], we found family history of diabetes to be an independent predictor of Type 2 diabetes in the full model. This finding may point to the strong association of Type 2 diabetes with genetic predisposition in the Iranian population. Similar to previous reports [16], some of the major components of the metabolic syndrome including abdominal obesity and hypertriglyceridemia independently predicted diabetes development in our population. This is important especially if one considers the increasing trend in prevalence of obesity in the TLGS population [23] and highlights the importance of preventing programs that target obesity control.

Like many other studies [13,15,16,19,24], we demonstrated that the strongest predictors of incident diabetes were abnormal glucose metabolism including IGT, IFG and in particular IFG/IGT. However, the risk associated with IFG was higher than that with IGT. This is in concordance with most European studies [13,15,17], but in contrast with the results of studies in Mauritians [25] and Asians [24] which showed higher risk in subjects with IGT. This could be due to the difference in the distribution of IFG and IGT by age and ethnicity as have been previously stated [26]. In the present study IFG/IGT had the highest associated risk with incident diabetes among other risk factors. This is most likely due to the fact that IFG and IGT have different pathophysiology and confirms previous report that combining 2 hPG and FPG significantly increases the power to discriminate those who develop diabetes from those who do not[26,27].

The current study has some limitations. First, we lost 30% of the original cohort in the follow-up examination. In addition, the comparison of risk profile at baseline between respondents and non-respondents showed higher values for some major diabetes risk factors in the former group which may cause overestimation. However, these differences, although statistically significant, did not seem to be clinically important. In addition, the mean values of FPG and 2 hPG as well as prevalence of IFG, IGT and IFG/IGT (data not shown), which were the strongest predictors of Type 2 diabetes, were not different in respondents and non-respondents. Second, we did not measure the association of physical activity with incident diabetes because our instruments used for assessing physical activity suffered from lack of accuracy as do any questionnaire used in epidemiological studies [28]. Third, there are uncertainties about generalizability of our results to ethnic groups other than those in the study and to other geographic regions of the country. The major strengths of the present study is being population based with large number of participants, using FPG and 2 hPG for detecting undiagnosed diabetes both at baseline and at follow-up, as well as adjusting for the major diabetes risk factors and confounders.

## Conclusion

In conclusion, Type 2 diabetes is increasing at a rate of nearly 1% per year in the Iranian urban population especially in those who are aged, obese or have abnormal glucose tolerance. Special attention in terms of preventive strategies must be paid to individuals with abnormal glucose tolerance, as this state was the most prominent predictor of developing type 2 diabetes.

## Competing interests

The authors declare that they have no competing interests.

## Authors' contributions

HH designed the study, performed the statistical analysis and drafted the manuscript. FH revised the manuscript critically for important intellectual content and gave final approval of the manuscript. NS and FA contributed to the conception and design of data. All authors read and approved the final manuscript.

## Pre-publication history

The pre-publication history for this paper can be accessed here:


